# Early Acute Spinal Subdural Hematoma Following Multilevel Bilateral Lumbar Decompression via a Unilateral Approach: A Case Report

**DOI:** 10.7759/cureus.94689

**Published:** 2025-10-15

**Authors:** Turgut Kuytu, Kudret Türeyen

**Affiliations:** 1 Neurosurgery, VM Medical Park Hospital, Bursa, TUR; 2 Neurosurgery, Private Practice, Bursa, TUR

**Keywords:** laminectomy, spinal decompression, spinal stenosis, spinal subdural hematoma, unilateral approach

## Abstract

We report a case diagnosed with lumbar spinal stenosis who underwent reoperation for a spinal subdural hematoma (SSH) following minimally invasive lumbar decompression in the early postoperative period. This study aimed to discuss the management of this rare complication.

A female patient with spinal stenosis underwent an uncomplicated left-sided, unilateral, three-level lumbar hemilaminectomy for bilateral decompression. However, six hours after surgery, she developed numbness and weakness in the right foot. An urgent lumbar MRI revealed an acute SSH at the operated levels. When paraparesis developed within minutes, the patient underwent emergency re-exploration. To our knowledge, this is the first reported case of SSH developing in the very early postoperative period.

SSH is a rare complication that should be considered in patients presenting with progressive neurological deficits after spinal surgery. Prompt evacuation of the hematoma remains the most appropriate treatment.

## Introduction

Spinal subdural hematomas (SSHs) typically occur in patients with vascular malformations, tumors, coagulation disorders, or those receiving anticoagulant therapy. They may also arise secondary to trauma, infection, or diagnostic/anesthetic puncture. However, SSHs rarely develop following spinal decompression surgery [1-3].

The number of reported cases of SSH secondary to surgical trauma is limited [1-5]. Consequently, little is known about the presentation and course of acute SSH after lumbar spine surgery. We aim to emphasize the need to better define potential risk factors, clinical presentation, management strategies, and associated morbidity of SSH, especially in cases where the established risk factors mentioned above are absent and when SSH develops very early after uncomplicated, minimally invasive spinal decompression surgery. Highlighting SSH in such cases underscores the importance of increased clinical awareness and timely intervention. Moreover, encouraging further reporting and retrospective analyses of SSH cases, particularly after minimally invasive and uncomplicated procedures, may help guide future clinical practice.

This article describes a patient with three-level degenerative lumbar spinal stenosis who underwent bilateral decompression through a left-sided unilateral approach and developed an early acute SSH. To our knowledge, this is the first reported case in the literature to present with symptoms in the very early postoperative period following unilateral decompression. We discuss the clinical and radiological findings, possible risk factors, complication management, and treatment considerations for this rare condition.

## Case presentation

A 70-year-old female patient with no significant medical history presented to the neurosurgery outpatient clinic with complaints of lower back pain and numbness radiating to both legs, which worsened during walking. Her walking distance had been limited to less than 25 m over the previous three months. Her past medical history included hypertension and diabetes mellitus, both well controlled with oral medication. Neurological examination findings were normal. Lumbosacral magnetic resonance imaging (MRI) revealed degenerative spinal stenosis at the L2-3, L3-4, and L4-5 levels.

As the patient did not respond to a course of conservative treatment, including pain medication and physical therapy, she underwent decompressive surgery consisting of a left-sided, three-level hemilaminectomy for bilateral nerve decompression. The procedure lasted two hours, with a total blood loss of 200 mL. No blood products were required, and no dural defect was observed intraoperatively.

**Figure 1:**
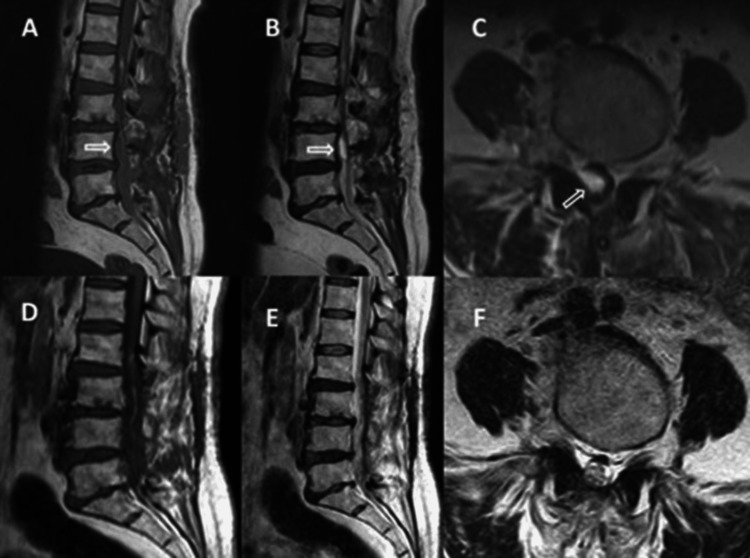
(A-C) Lumbar magnetic resonance images of the subdural hematoma. (D-F) Control lumbar magnetic resonance images one year after the surgery. (A) Isointense hematoma on sagittal T1-weighted image. (B) Hyperintense hematoma on sagittal T2-weighted image. (C) Hyperintense hematoma on axial T2-weighted image. (D) Sagittal T1-weighted image. (E) Sagittal T2-weighted image. (F) Axial T2-weighted image.

Early postoperative findings were unremarkable. However, six hours after surgery, the patient complained of numbness in her right leg, followed within 20 minutes by weakness in her right foot. An emergency lumbosacral MRI was immediately performed. Shortly after the scan, she developed hypoesthesia in the right L2-L5 dermatomes and progressive paraparesis, with Grade 3 weakness more pronounced on the right side. No abnormal blood accumulation was detected in the deep hemovac drain. MRI revealed an isointense lesion on sagittal T1-weighted images and a hyperintense hematoma on sagittal T2-weighted sections at the L4 level, consistent with an acute SSH. Given the patient’s acute motor neurological deterioration, emergency surgical exploration was performed (Figures 1A-1C).

**Figure 2:**
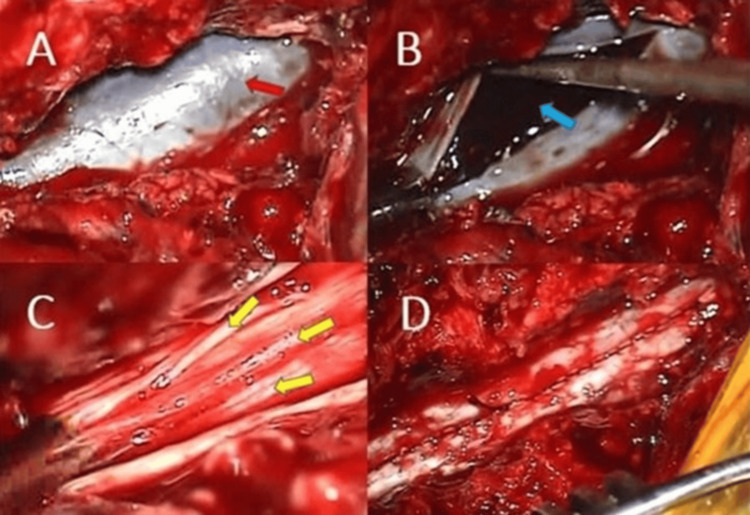
Intraoperative appearance of the spinal subdural hematoma. (A) The dura mater is stretched and dark in color before dural opening, reflecting the presence of blood beneath it (red arrow: dura mater). (B) Appearance of the organized subdural hematoma after dural opening (blue arrow: organized subdural hematoma). (C) Nerve rootlets after hematoma evacuation (yellow arrows: rootlets). (D) Appearance of the dura following watertight closure.

The previous skin incision was reopened, and the surgical microscope was brought into the operative field. Under microscopic visualization, the prior L2-4 hemilaminectomies were extended to expose the dura. The dura appeared stretched and dark purple in color (Figure 2A). No organized hematoma or significant compressive mass was identified in the epidural space. The dura was then opened, revealing an underlying organized subdural hematoma (Figure 2B). The hematoma was carefully removed using microdissectors and an aspirator. Following evacuation, the spinal cord was decompressed, the nerve rootlets were freed, and cerebrospinal fluid (CSF) flow was re-established. The subdural space appeared unremarkable, with no evidence of active bleeding or any pathology responsible for the hemorrhage (Figure 2C). The dura was then closed in a watertight manner (Figure 2D).

In the early postoperative period, neurological examination revealed bilateral paraparesis with a muscle strength of 2/5. Postoperative MRI confirmed complete evacuation of the hematoma. Three weeks after surgery, the patient achieved full recovery from paraparesis following a course of physical therapy. To our knowledge, this is the first reported case of spinal subdural hematoma developing in the very early postoperative period after surgery.

## Discussion

SSH is an extremely rare complication of spine surgery, with only a few cases reported to date. In 1993, Reinsel et al. described a case of SSH that developed six weeks after surgery for recurrent lumbar disc herniation [3]. They suggested that a traumatic dural puncture during myelography or iatrogenic dural trauma during discectomy could have been the cause [3]. Gehri et al. later reported a case of lumbar discectomy in which the patient developed symptomatic acute SSH on postoperative day 6 due to an intraoperative dural tear that had been repaired successfully during surgery [2]. Similarly, that report identified intraoperative dural injury as a possible etiologic factor [2]. Chang et al. presented a case following lumbar posterior segmental instrumentation and emphasized that SSH may occur even in patients without any known predisposing factors [1]. Lykissas et al. [4] described two cases of SSH: one patient, on anticoagulation therapy for coronary artery disease, developed urinary difficulty two weeks after decompressive laminectomy for spinal stenosis, which resolved with conservative management. The second patient sustained an unintentional dural injury during bilateral L3-4 laminectomy and required re-exploration on postoperative day 2 due to severe bilateral leg pain and urinary retention unresponsive to medical therapy. In 2017, Boe et al. reported a 76-year-old man with central and lateral recess spinal stenosis who underwent an apparently uncomplicated bilateral L3-4 and left-sided L4-5 decompressive partial laminectomy and discectomy. On postoperative day 2, he developed progressive lower extremity weakness, sensory changes, and urinary retention. Thoracolumbar MRI revealed subdural compression at the T11-L2 levels, necessitating emergency evacuation of the hematoma [5].

The present case involved multilevel bilateral decompression of the spinal cord performed via a unilateral approach. The patient had no known predisposing factors for SSH, such as vascular malformation, tumor, coagulopathy, preoperative anticoagulant therapy, trauma, or dural tear or puncture. To verify the absence of inadvertent dural penetration during surgery, the operative video was reviewed. However, the patient did have less common risk factors for SSH, namely, diabetes mellitus and hypertension, as noted in some previous reports [6]. In this context, even in the absence of established prognostic factors, such complications should be considered in patients with controlled hypertension and diabetes mellitus. Additionally, in elderly patients, bleeding may be more likely due to an increased subdural space secondary to age-related atrophy, similar to that observed in the brain.

Acute SSH may present with a range of clinical findings depending on the hematoma’s location and the extent of neural tissue compression and tolerance. At the lumbar level, SSH has been associated with severe lower back pain and radiculopathy radiating to the legs, along with varying degrees of motor, sensory, and reflex deficits. Cauda equina syndrome may also occur, resulting in urinary and fecal incontinence [2,3].

Because SSH can lead to progressive and potentially permanent neurological deficits, prompt recognition and emergency intervention are often required. Therefore, early diagnosis is crucial. In our case, close neurological monitoring during the early postoperative period was the key factor that enabled rapid identification and management of this complication.

Furthermore, "emergency decompression surgery" to evacuate the hematoma after diagnosis can reverse impending but not yet complete neural injury. In our case, rapid neurological recovery was observed following decompression and physiotherapy, with the patient’s 2/5 paraparesis fully resolving within approximately three weeks. Thus, the timing of surgical intervention is crucial. Prompt neural decompression following clinical deterioration and rapid diagnosis can prevent potentially permanent neurological damage.

MRI remains the preferred imaging modality for diagnosis, as it clearly demonstrates the subdural hematoma extending along the dural boundary on axial sections. This feature is critical for differentiating a subdural hematoma from an epidural one, as the latter typically causes external compression with an irregular dural boundary [1]. Depending on the hematoma’s stage, MRI findings may vary in signal intensity [1]. For example, a hyperacute subdural hematoma may appear isointense on T1-weighted images and hyperintense on T2-weighted images. In the present case, the acute subdural hematoma appeared isointense on T1-weighted images and hyperintense on T2-weighted images (Figures 1A-1C).

## Conclusions

In conclusion, an acute SSH is a rare pathology that should be considered, especially for cases showing progressive neurologic deficit following spinal surgery. Routine close monitoring of the patient’s leg movements during the postoperative period until mobilization may prevent delays in identifying the pathology. MRI has been the primary method for diagnosis. Especially in cases with progressive neurologic deficit, emergency hematoma evacuation as early as possible is the most appropriate treatment method.
